# Poly[[diaqua­(3-carb­oxy-5-nitro­ben­zo­ato)(μ-5-nitro­benzene-1,3-dicarboxyl­ato)neodymium(III)] 2.5-hydrate]

**DOI:** 10.1107/S1600536809032486

**Published:** 2009-08-22

**Authors:** Nurul-Nadia Raime, Rohana Adnan, Mohd Mustaqim Rosli, Hoong-Kun Fun

**Affiliations:** a School of Chemical Sciences, Universiti Sains Malaysia, 11800 USM, Penang, Malaysia; b X-ray Crystallography Unit, School of Physics, Universiti Sains Malaysia, 11800 USM, Penang, Malaysia

## Abstract

In the title compound, {[Nd(C_8_H_3_NO_6_)(C_8_H_4_NO_6_)(H_2_O)_2_]·2.5H_2_O}_*n*_, the Nd^II^ ion is nine-coordinated by seven O atoms from five carboxyl­ate groups and two water mol­ecules. The [Nd(C_8_H_3_NO_6_)(H_2_O)_2_]^2+^ units are bridged by 5-nitro­isophthalate dianions, forming polymeric sheets parallel to the *ab* plane. The polymeric sheets are linked into a three-dimensional network by O—H⋯O and C—H⋯O hydrogen bonds, and π–π inter­actions [centroid–centroid distance = 3.5533 (11) Å]. The 5-nitro­isophthalate(1−) anion is disordered over three positions with an occupancy ratio of 0.68:0.23:0.09. Two of the uncoordinated water mol­ecules are disordered over two positions, with occupancy ratios of 0.722 (15):0.278 (15) and 0.279 (6):0.221 (6), respectively.

## Related literature

For related structures, see: Ye *et al.* (2008 ▶); Eddaoudi *et al.* (2001 ▶); Bünzli & Choppin (1989 ▶); Huang *et al.* (2008 ▶); Cui *et al.* (2002 ▶); Yan *et al.* (2005 ▶); Ren *et al.* (2006 ▶); Li *et al.* (2005 ▶). For the stability of the temperature controller used for the data collection, see: Cosier & Glazer (1986 ▶).
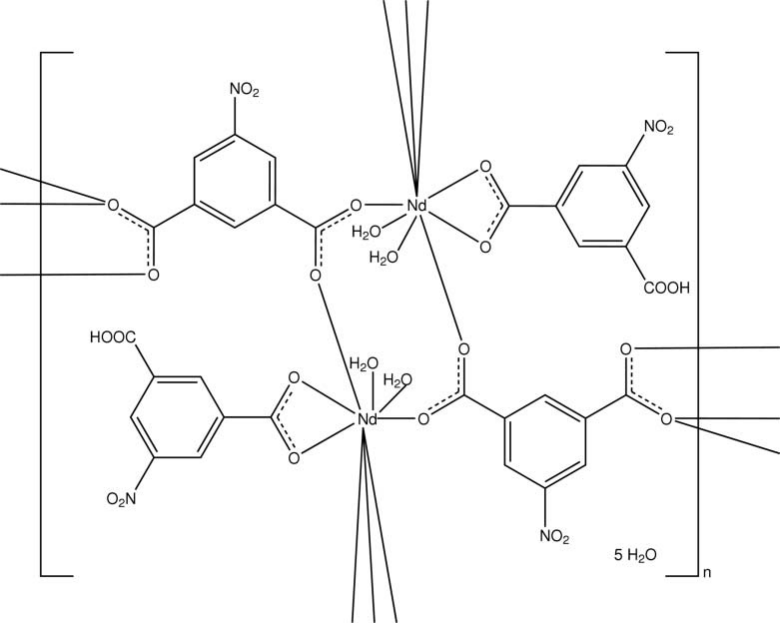


## Experimental

### 

#### Crystal data

[Nd(C_8_H_3_NO_6_)(C_8_H_4_NO_6_)(H_2_O)_2_]·2.5H_2_O
*M* *_r_* = 644.55
Triclinic, 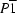

*a* = 9.5748 (1) Å
*b* = 10.4634 (1) Å
*c* = 13.3285 (2) Å
α = 69.279 (1)°
β = 71.753 (1)°
γ = 66.046 (1)°
*V* = 1118.71 (2) Å^3^
*Z* = 2
Mo *K*α radiation
μ = 2.41 mm^−1^
*T* = 100 K
0.35 × 0.24 × 0.13 mm

#### Data collection

Bruker SMART APEXII CCD area-detector diffractometer
Absorption correction: multi-scan (*SADABS*; Bruker, 2005 ▶) *T*_min_ = 0.484, *T*_max_ = 0.742
22527 measured reflections
5879 independent reflections
5725 reflections with *I* > 2σ(*I*)
*R*_int_ = 0.019

#### Refinement

*R*[*F* ^2^ > 2σ(*F* ^2^)] = 0.018
*wR*(*F* ^2^) = 0.057
*S* = 1.21
5879 reflections
398 parameters
115 restraints
H-atom parameters constrained
Δρ_max_ = 1.18 e Å^−3^
Δρ_min_ = −0.77 e Å^−3^

### 

Data collection: *APEX2* (Bruker, 2005 ▶); cell refinement: *SAINT* (Bruker, 2005 ▶); data reduction: *SAINT*; program(s) used to solve structure: *SHELXTL* (Sheldrick, 2008 ▶); program(s) used to refine structure: *SHELXTL*; molecular graphics: *SHELXTL*; software used to prepare material for publication: *SHELXTL* and *PLATON* (Spek, 2009 ▶).

## Supplementary Material

Crystal structure: contains datablocks global, I. DOI: 10.1107/S1600536809032486/ci2868sup1.cif

Structure factors: contains datablocks I. DOI: 10.1107/S1600536809032486/ci2868Isup2.hkl

Additional supplementary materials:  crystallographic information; 3D view; checkCIF report

## Figures and Tables

**Table 1:** Selected bond lengths (Å)

Nd1—O7	2.3302 (13)
Nd1—O8^i^	2.4214 (14)
Nd1—O10^ii^	2.4481 (14)
Nd1—O1*W*	2.4612 (14)
Nd1—O9^iii^	2.4708 (13)
Nd1—O2*W*	2.4775 (15)
Nd1—O2	2.5223 (13)
Nd1—O1	2.5743 (14)
Nd1—O10^iii^	2.9332 (14)

Symmetry codes: (i) 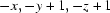; (ii) 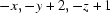; (iii) 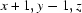.

**Table 2:** Hydrogen-bond geometry (Å, °)

*D*—H⋯*A*	*D*—H	H⋯*A*	*D*⋯*A*	*D*—H⋯*A*
O5*A*—H5*A*⋯O3*W*^iv^	0.84	1.76	2.596 (5)	178
O1*W*—H1*W*1⋯O2^v^	0.74	1.99	2.731 (3)	175
O1*W*—H2*W*1⋯O4*WA*	0.94	2.00	2.849 (4)	149
O2*W*—H1*W*2⋯O4*WA*	0.84	1.86	2.667 (3)	159
O2*W*—H2*W*2⋯O1^i^	0.82	2.06	2.874 (3)	172
O3*W*—H1*W*3⋯O11^vi^	0.91	2.56	3.246 (3)	132
O3*W*—H1*W*3⋯O12^vi^	0.91	2.05	2.902 (3)	156
O3*W*—H2*W*3⋯O9^iii^	0.96	1.76	2.688 (2)	162
O4*WA*—H2*W*4⋯O3*W*	0.85	2.51	3.118 (6)	130
C12—H12*A*⋯O1^ii^	0.95	2.41	3.351 (2)	169

Symmetry codes: (i) 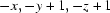; (ii) 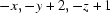; (iii) 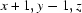; (iv) 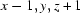; (v) 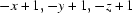; (vi) 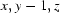.

**Fig. 1.**
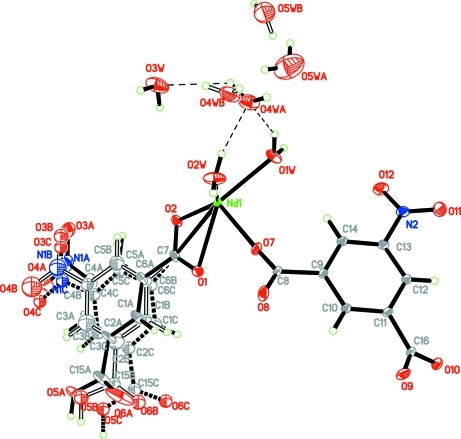
The asymetric unit of the title compound. Displacement ellipsoids are drawn at the 50% probability level. All disorder components are shown. Hydrogen bonds are shown as dashed lines.

**Fig. 2.**
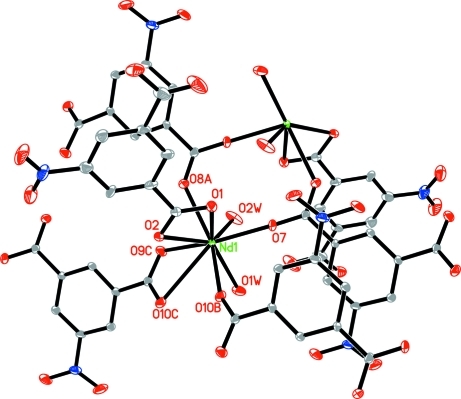
Part of the polymeric sheet, showing the coordination environment of NdIII ion. Displacement ellipsoids are drawn at the 50% probability level. Only major disorder components are shown. Uncoordinated water molecules and H atoms have been omitted for clarity. Symmetry codes: (A) -x, 1-y, 1-z ; (B) -x, 2-y, 1-z; (c) 1+x, -1+y, z.

**Fig. 3.**
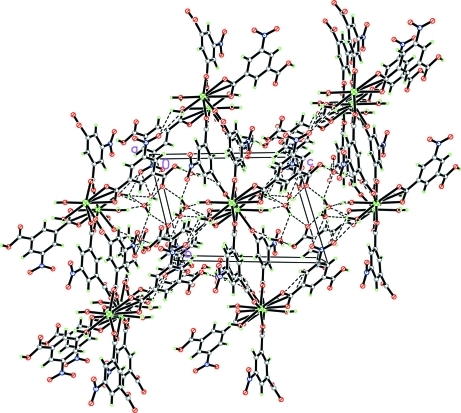
The crystal packing of the title compound, viewed down the a axis. Hydrogen bonds are shown as dashed lines. Only major disorder components are shown.

## supplementary crystallographic information

### Comment

The research on the assembly of lanthanide coordination networks has increased over the years (Ye *et al.*, 2008; Eddaoudi *et al.*, 2001). Lanthanide provide opportunities for the discovery of unusual network topologies (Bünzli & Choppin, 1989; Huang *et al.*, 2008; Cui *et al.*, 2002) due to its high and variable coordination numbers and flexible coordination environments (Yan *et al.*, 2005).

5- Nitroisophthalic acid (nia) has two carboxylic groups which may be completely or partially deprotonated and thus produces versatile coordination modes with lanthanide ions. Moreover, the existence of nitro group as an electron-withdrawing group has profound impacts on the electron density of the whole ligand, and thereby different physical phenomena can be produced (Ren *et al.*, 2006; Li *et al.*, 2005). In this paper, we report the crystal structure of a polymeric coordination complex formed from hydrothermal reaction between trivalent neodymium ion and nitroisophthalic acid.

The asymmetric unit of the title polymeric compound is shown in Fig. 1. In the crystal structure, each Nd^III^ ion adopts a nine-coordination environment being coordinated by two O atoms from two water molecules and seven O atoms from five carboxylate groups. The Nd—O distances range from 2.3302 (13) to 2.9332 (14) Å for carboxylate groups and 2.4612 (14) and 2.4775 (15) Å, respectively for O1W and O2W (Table 1). The nitro group attached to the C1-C6 benzene ring is slightly twisted, with a dihedral angle of 12.2 (3)° for the major disorder component A [ 9(1)° for B and 3(1)° for C]. The nitro group attached to the C9-C14 benzene ring is twisted by a dihedral angle of 21.2 (1)°. The dihedral angles between C1-C6 benzene rings and planes of carboxyl groups in disorder components A, B and C are 5.8 (5)°, 9(1)° and 29 (1)°, respectively. The adjacent [Nd(C_8_H_3_NO_6_)(H_2_O)_2_]^2+^ units are bridged by 5-nitroisophthalate dianions to form polymeric sheets parallel to the *ab* plane (Fig. 2).

The polymeric sheets are linked into a three-dimensional network (Fig. 3) by O—H···O and C—H···O hydrogen bonds (Table 2). There also exist a π–π interaction between the C9–C14 benzene rings at (x, y, z) and (-x, 2-y, 1-z), with a centroid-to-centroid distance of 3.5533 (11) Å.

### Experimental

A mixture of 5-nitroisophthalic acid (0.4243 g, 2.0 mmol), sodium hydroxide (0.140 g, 3.5 mmol) and distilled water (30 ml) was heated till boiling. The solution was left to cool to room temperature before neodymium(III) nitrate hexahydrate salt, Nd(NO_3_).6H_2_O (0.4380 g, 1.0 mmol) was added to it. The pH of the mixture was controlled to be between 3–4 by adding 1 *M* NaOH or 1 *M* HNO_3_. Subsequently the solution was poured into a 40 ml Teflon tube, which was then sealed and heated at 403 K for 3 d. Upon cooling to room temperature, light purple crystals of the title compound were obtained which were filtered, washed with distilled water and left to dry in air.

### Refinement

The 5-nitroisophthalate(1-) anion is disordered over three positions with an occupancy ratio of 0.683 (7):0.234 (7):0.087 (7) which was fixed at 0.68:0.23:0.09 for the final refinement. The two minor disorder components B and C were refined isotropically; a common *U*_iso_ was used for the disorder component C. In all disorder components, the C1—C6 benzene ring is constrained to a regular hexagon with d = 1.39 Å. Atom O4W and hemihydrate O5W are also disordered over two positions, with occupancies of 0.722 (15) and 0.278 (15), and 0.279 (6) and 0.221 (6) respectively. For the disordered 5-nitroisophthalate(1-) anion, similarity restraints were applied. The O atoms of the uncoordinated water molecules are restrained so that their U_ij_ components approximate to isotropic behavior. H atoms for O1W, O2W, O3W and O4WB were located in a difference Fourier map and refined as riding with the parent atom with *U*_iso_(H) = 1.5*U*_eq_(O). The rest of H atoms were positioned geometrically and refined using a riding model, with C-H = 0.95 Å, O-H = 0.84 Å and *U*_iso_ = 1.2_eq_(C) and 1.5_eq_(O).

### Crystal data

**Table:**

[Nd(C_8_H_3_NO_6_)(C_8_H_4_NO_6_)(H_2_O)_2_]·2.5H_2_O	*Z* = 2
*M**_r_* = 644.55	*F*(000) = 636
Triclinic, *P*1	*D*_x_ = 1.913 Mg m^−^^3^
Hall symbol: -P 1	Mo *K*α radiation, λ = 0.71073 Å
*a* = 9.5748 (1) Å	Cell parameters from 9930 reflections
*b* = 10.4634 (1) Å	θ = 2.7–29.0°
*c* = 13.3285 (2) Å	µ = 2.41 mm^−^^1^
α = 69.279 (1)°	*T* = 100 K
β = 71.753 (1)°	Plate, purple
γ = 66.046 (1)°	0.35 × 0.24 × 0.13 mm
*V* = 1118.71 (2) Å^3^	

### Data collection

**Table:**

Bruker SMART APEXII CCD area-detector diffractometer	5879 independent reflections
Radiation source: fine-focus sealed tube	5725 reflections with *I* > 2σ(*I*)
graphite	*R*_int_ = 0.019
φ and ω scans	θ_max_ = 29.0°, θ_min_ = 1.7°
Absorption correction: multi-scan (*SADABS*; Bruker, 2005)	*h* = −13→13
*T*_min_ = 0.484, *T*_max_ = 0.742	*k* = −14→14
22527 measured reflections	*l* = −18→18

### Refinement

**Table:**

Refinement on *F*^2^	Primary atom site location: structure-invariant direct methods
Least-squares matrix: full	Secondary atom site location: difference Fourier map
*R*[*F*^2^ > 2σ(*F*^2^)] = 0.018	Hydrogen site location: inferred from neighbouring sites
*wR*(*F*^2^) = 0.057	H-atom parameters constrained
*S* = 1.21	*w* = 1/[σ^2^(*F*_o_^2^) + (0.0337*P*)^2^ + 0.5091*P*] where *P* = (*F*_o_^2^ + 2*F*_c_^2^)/3
5879 reflections	(Δ/σ)_max_ = 0.001
398 parameters	Δρ_max_ = 1.18 e Å^−^^3^
115 restraints	Δρ_min_ = −0.77 e Å^−^^3^

### Special details

**Table:**

Experimental. The crystal was placed in the cold stream of an Oxford Cyrosystems Cobra open-flow nitrogen cryostat (Cosier & Glazer, 1986) operating at 100.0 (1) K.
Geometry. All e.s.d.'s (except the e.s.d. in the dihedral angle between two l.s. planes) are estimated using the full covariance matrix. The cell e.s.d.'s are taken into account individually in the estimation of e.s.d.'s in distances, angles and torsion angles; correlations between e.s.d.'s in cell parameters are only used when they are defined by crystal symmetry. An approximate (isotropic) treatment of cell e.s.d.'s is used for estimating e.s.d.'s involving l.s. planes.
Refinement. Refinement of *F*^2^ against ALL reflections. The weighted *R*-factor *wR* and goodness of fit *S* are based on *F*^2^, conventional *R*-factors *R* are based on *F*, with *F* set to zero for negative *F*^2^. The threshold expression of *F*^2^ > σ(*F*^2^) is used only for calculating *R*-factors(gt) *etc*. and is not relevant to the choice of reflections for refinement. *R*-factors based on *F*^2^ are statistically about twice as large as those based on *F*, and *R*- factors based on ALL data will be even larger.

### Fractional atomic coordinates and isotropic or equivalent isotropic displacement parameters (Å^2^)

**Table:**

	*x*	*y*	*z*	*U*_iso_*/*U*_eq_	Occ. (<1)
Nd1	0.285572 (9)	0.468414 (9)	0.486911 (7)	0.01037 (4)	
O3A	0.5272 (5)	−0.1271 (4)	0.9533 (3)	0.0330 (9)	0.68
O4A	0.3769 (4)	−0.1558 (3)	1.1135 (2)	0.0463 (8)	0.68
O5A	−0.1663 (5)	0.1114 (5)	1.1553 (3)	0.0263 (8)	0.68
H5A	−0.2601	0.1301	1.1875	0.039*	0.68
O6A	−0.2644 (3)	0.3037 (5)	1.0252 (3)	0.0605 (13)	0.68
N1A	0.4039 (6)	−0.0919 (4)	1.0164 (3)	0.0297 (7)	0.68
C1A	0.0383 (3)	0.2754 (4)	0.8934 (3)	0.0205 (8)	0.68
H1AA	−0.0439	0.3572	0.8654	0.025*	0.68
C2A	0.0078 (3)	0.1835 (5)	0.9962 (3)	0.0198 (8)	0.68
C3A	0.1281 (4)	0.0639 (4)	1.0372 (2)	0.0238 (9)	0.68
H3AA	0.1072	0.0011	1.1075	0.029*	0.68
C4A	0.2789 (4)	0.0361 (3)	0.9754 (3)	0.0245 (8)	0.68
C5A	0.3094 (3)	0.1279 (4)	0.8726 (3)	0.0189 (9)	0.68
H5AA	0.4125	0.1089	0.8304	0.023*	0.68
C6A	0.1891 (4)	0.2476 (4)	0.8316 (3)	0.0165 (13)	0.68
C15A	−0.1548 (5)	0.2079 (7)	1.0602 (3)	0.0268 (9)	0.68
O3B	0.4896 (15)	−0.1413 (17)	0.9604 (13)	0.027 (3)*	0.23
O4B	0.3076 (14)	−0.1937 (12)	1.0907 (9)	0.056 (3)*	0.23
O5B	−0.1948 (16)	0.1468 (13)	1.1445 (11)	0.025 (3)*	0.23
H5B	−0.2852	0.1742	1.1814	0.037*	0.23
O6B	−0.2641 (10)	0.3699 (8)	1.0410 (7)	0.0257 (18)*	0.23
N1B	0.3557 (15)	−0.1115 (12)	1.0079 (10)	0.040 (3)*	0.23
C1B	0.0367 (14)	0.3066 (10)	0.8974 (11)	0.016 (3)*	0.23
H1BA	−0.0349	0.4004	0.8737	0.019*	0.23
C2B	−0.0066 (12)	0.2128 (14)	0.9957 (11)	0.038 (5)*	0.23
C3B	0.0982 (14)	0.0756 (13)	1.0304 (10)	0.039 (5)*	0.23
H3BA	0.0686	0.0115	1.0976	0.046*	0.23
C4B	0.2463 (12)	0.0322 (10)	0.9668 (10)	0.021 (3)*	0.23
C5B	0.2896 (12)	0.1260 (15)	0.8684 (10)	0.028 (4)*	0.23
H5BA	0.3907	0.0963	0.8249	0.033*	0.23
C6B	0.1848 (16)	0.2632 (14)	0.8337 (10)	0.016 (4)*	0.23
C15B	−0.1695 (13)	0.2567 (12)	1.0630 (10)	0.019 (3)*	0.23
O3C	0.449 (2)	−0.130 (3)	0.9824 (19)	0.0208 (13)*	0.09
O4C	0.272 (2)	−0.1538 (18)	1.1276 (12)	0.0208 (13)*	0.09
O5C	−0.2380 (19)	0.2386 (18)	1.1423 (12)	0.0208 (13)*	0.09
H5C	−0.3302	0.2675	1.1873	0.025*	0.09
O6C	−0.2201 (18)	0.4585 (14)	1.0575 (13)	0.0208 (13)*	0.09
N1C	0.318 (2)	−0.0826 (18)	1.0374 (14)	0.0208 (13)*	0.09
C1C	0.0294 (19)	0.3429 (15)	0.9028 (13)	0.0208 (13)*	0.09
H1CA	−0.0355	0.4382	0.8740	0.025*	0.09
C2C	−0.0198 (15)	0.2637 (16)	1.0071 (12)	0.0208 (13)*	0.09
C3C	0.0750 (18)	0.1244 (16)	1.0493 (11)	0.0208 (13)*	0.09
H3CA	0.0414	0.0702	1.1206	0.025*	0.09
C4C	0.2191 (19)	0.0642 (16)	0.9873 (14)	0.0208 (13)*	0.09
C5C	0.2683 (19)	0.143 (2)	0.8830 (15)	0.0208 (13)*	0.09
H5CA	0.3668	0.1023	0.8406	0.025*	0.09
C6C	0.173 (2)	0.283 (2)	0.8408 (12)	0.0208 (13)*	0.09
C15C	−0.174 (2)	0.3312 (17)	1.0721 (15)	0.0208 (13)*	0.09
O1	0.11719 (16)	0.45727 (15)	0.68090 (11)	0.0170 (3)	
O2	0.36376 (16)	0.31566 (15)	0.66785 (11)	0.0165 (3)	
O7	0.05148 (16)	0.65931 (14)	0.46945 (12)	0.0176 (3)	
O8	−0.19586 (17)	0.73727 (15)	0.45331 (13)	0.0209 (3)	
O9	−0.49580 (16)	1.26834 (15)	0.42188 (12)	0.0188 (3)	
O10	−0.37511 (16)	1.38214 (14)	0.45852 (11)	0.0157 (3)	
O11	0.1860 (2)	1.24682 (18)	0.22910 (16)	0.0325 (4)	
O12	0.32156 (17)	1.02380 (18)	0.29539 (14)	0.0253 (3)	
N2	0.19668 (19)	1.12233 (19)	0.28241 (14)	0.0178 (3)	
C7	0.2251 (2)	0.3495 (2)	0.72073 (15)	0.0155 (3)	
C8	−0.0736 (2)	0.75718 (19)	0.44717 (14)	0.0122 (3)	
C9	−0.0762 (2)	0.91156 (18)	0.41124 (14)	0.0107 (3)	
C10	−0.2178 (2)	1.02243 (19)	0.42723 (14)	0.0115 (3)	
H10A	−0.3122	1.0009	0.4554	0.014*	
C11	−0.2213 (2)	1.16466 (19)	0.40205 (14)	0.0113 (3)	
C12	−0.0849 (2)	1.19940 (19)	0.35355 (14)	0.0124 (3)	
H12A	−0.0861	1.2963	0.3343	0.015*	
C13	0.0528 (2)	1.0867 (2)	0.33446 (14)	0.0129 (3)	
C14	0.0619 (2)	0.94284 (19)	0.36509 (14)	0.0124 (3)	
H14A	0.1593	0.8681	0.3549	0.015*	
C16	−0.3728 (2)	1.27944 (19)	0.42875 (14)	0.0127 (3)	
O1W	0.39421 (17)	0.62127 (17)	0.31765 (11)	0.0214 (3)	
H1W1	0.4595	0.6422	0.3181	0.032*	
H2W1	0.4207	0.6070	0.2475	0.032*	
O2W	0.20344 (18)	0.4620 (2)	0.33051 (13)	0.0285 (4)	
H1W2	0.2599	0.4648	0.2674	0.043*	
H2W2	0.1146	0.4785	0.3245	0.043*	
O3W	0.5412 (2)	0.1731 (2)	0.24946 (15)	0.0377 (4)	
H1W3	0.4742	0.1364	0.2425	0.057*	
H2W3	0.5221	0.1903	0.3193	0.057*	
O4WA	0.4139 (3)	0.5063 (6)	0.1473 (2)	0.0342 (12)	0.722 (15)
H1W4	0.3780	0.5660	0.0913	0.051*	0.722 (15)
H2W4	0.4716	0.4275	0.1301	0.051*	0.722 (15)
O4WB	0.4055 (9)	0.4415 (15)	0.1312 (8)	0.037 (3)	0.278 (15)
H3W4	0.3704	0.4757	0.0809	0.056*	0.278 (15)
H4W4	0.4353	0.3561	0.1412	0.056*	0.278 (15)
O5WA	0.6107 (14)	0.5930 (14)	0.0951 (10)	0.082 (4)	0.279 (6)
H1W5	0.7059	0.5714	0.0630	0.123*	0.279 (6)
H2W5	0.5861	0.5169	0.1155	0.123*	0.279 (6)
O5WB	0.9195 (14)	0.4364 (12)	0.0923 (8)	0.051 (3)	0.221 (6)
H3W5	0.8615	0.4864	0.1375	0.077*	0.221 (6)
H4W5	1.0037	0.3835	0.1152	0.077*	0.221 (6)

### Atomic displacement parameters (Å^2^)

**Table:**

	*U*^11^	*U*^22^	*U*^33^	*U*^12^	*U*^13^	*U*^23^
Nd1	0.00707 (6)	0.00691 (6)	0.01558 (6)	−0.00155 (4)	−0.00089 (3)	−0.00326 (4)
O3A	0.038 (2)	0.0212 (16)	0.0264 (15)	−0.0010 (16)	−0.0073 (15)	−0.0004 (11)
O4A	0.064 (2)	0.0291 (15)	0.0201 (12)	−0.0069 (14)	−0.0055 (13)	0.0107 (10)
O5A	0.0263 (18)	0.037 (2)	0.0144 (13)	−0.0199 (18)	0.0051 (12)	−0.0027 (14)
O6A	0.0223 (14)	0.103 (3)	0.0277 (14)	−0.0250 (18)	−0.0064 (11)	0.0225 (18)
N1A	0.043 (2)	0.0183 (15)	0.0194 (14)	−0.0077 (16)	−0.0092 (15)	0.0041 (11)
C1A	0.0177 (17)	0.027 (2)	0.0192 (16)	−0.0148 (15)	−0.0041 (11)	−0.0003 (16)
C2A	0.0189 (16)	0.034 (2)	0.0095 (14)	−0.0193 (14)	−0.0011 (10)	0.0016 (13)
C3A	0.036 (2)	0.0240 (19)	0.0128 (14)	−0.0182 (16)	−0.0061 (15)	0.0037 (11)
C4A	0.035 (2)	0.0231 (19)	0.0186 (16)	−0.0142 (16)	−0.0103 (16)	0.0006 (12)
C5A	0.0233 (17)	0.0150 (17)	0.0155 (15)	−0.0079 (14)	−0.0039 (13)	0.0010 (11)
C6A	0.018 (2)	0.019 (2)	0.0147 (17)	−0.0128 (14)	−0.0026 (10)	0.0009 (12)
C15A	0.0245 (18)	0.044 (3)	0.0150 (16)	−0.0223 (19)	−0.0037 (12)	0.0009 (17)
O1	0.0127 (6)	0.0151 (6)	0.0183 (6)	−0.0033 (5)	−0.0015 (5)	−0.0018 (5)
O2	0.0112 (6)	0.0155 (6)	0.0192 (6)	−0.0062 (5)	−0.0027 (5)	0.0014 (5)
O7	0.0123 (6)	0.0091 (6)	0.0256 (7)	0.0005 (5)	−0.0037 (5)	−0.0026 (5)
O8	0.0148 (7)	0.0110 (6)	0.0374 (8)	−0.0051 (5)	−0.0068 (6)	−0.0047 (6)
O9	0.0096 (6)	0.0161 (7)	0.0311 (7)	−0.0019 (5)	−0.0015 (5)	−0.0117 (6)
O10	0.0170 (6)	0.0099 (6)	0.0203 (6)	−0.0039 (5)	−0.0009 (5)	−0.0072 (5)
O11	0.0217 (8)	0.0195 (8)	0.0512 (10)	−0.0135 (6)	0.0080 (7)	−0.0089 (7)
O12	0.0092 (6)	0.0269 (8)	0.0378 (8)	−0.0053 (6)	0.0001 (6)	−0.0111 (7)
N2	0.0120 (7)	0.0190 (8)	0.0244 (8)	−0.0080 (6)	0.0022 (6)	−0.0099 (6)
C7	0.0139 (8)	0.0152 (9)	0.0172 (8)	−0.0072 (7)	−0.0030 (6)	−0.0014 (6)
C8	0.0118 (8)	0.0086 (7)	0.0145 (7)	−0.0024 (6)	−0.0007 (6)	−0.0040 (6)
C9	0.0091 (7)	0.0079 (7)	0.0143 (7)	−0.0014 (6)	−0.0028 (6)	−0.0030 (6)
C10	0.0081 (7)	0.0107 (8)	0.0150 (7)	−0.0027 (6)	−0.0014 (6)	−0.0039 (6)
C11	0.0085 (7)	0.0097 (8)	0.0148 (7)	−0.0014 (6)	−0.0018 (6)	−0.0048 (6)
C12	0.0111 (8)	0.0109 (8)	0.0155 (7)	−0.0041 (6)	−0.0008 (6)	−0.0048 (6)
C13	0.0094 (8)	0.0139 (8)	0.0168 (7)	−0.0057 (6)	0.0003 (6)	−0.0059 (6)
C14	0.0086 (7)	0.0121 (8)	0.0155 (7)	−0.0023 (6)	−0.0013 (6)	−0.0048 (6)
C16	0.0113 (8)	0.0089 (8)	0.0155 (7)	−0.0024 (6)	−0.0001 (6)	−0.0038 (6)
O1W	0.0170 (7)	0.0319 (8)	0.0164 (6)	−0.0145 (6)	−0.0027 (5)	−0.0008 (6)
O2W	0.0138 (7)	0.0522 (11)	0.0270 (7)	−0.0106 (7)	−0.0007 (6)	−0.0222 (7)
O3W	0.0305 (9)	0.0669 (13)	0.0304 (8)	−0.0311 (9)	0.0039 (7)	−0.0205 (9)
O4WA	0.0301 (14)	0.046 (3)	0.0198 (11)	−0.0101 (13)	−0.0026 (9)	−0.0065 (11)
O4WB	0.027 (3)	0.045 (5)	0.030 (3)	−0.013 (3)	0.005 (3)	−0.005 (4)
O5WA	0.072 (6)	0.091 (7)	0.103 (7)	−0.024 (5)	−0.012 (5)	−0.058 (6)
O5WB	0.063 (6)	0.052 (6)	0.045 (5)	−0.022 (5)	−0.011 (4)	−0.017 (4)

### Geometric parameters (Å, °)

**Table:**

Nd1—O7	2.3302 (13)	C1C—C2C	1.39
Nd1—O8^i^	2.4214 (14)	C1C—C6C	1.39
Nd1—O10^ii^	2.4481 (14)	C1C—H1CA	0.95
Nd1—O1W	2.4612 (14)	C2C—C3C	1.39
Nd1—O9^iii^	2.4708 (13)	C2C—C15C	1.486 (13)
Nd1—O2W	2.4775 (15)	C3C—C4C	1.39
Nd1—O2	2.5223 (13)	C3C—H3CA	0.95
Nd1—O1	2.5743 (14)	C4C—C5C	1.39
Nd1—C7	2.8822 (18)	C5C—C6C	1.39
Nd1—O10^iii^	2.9332 (14)	C5C—H5CA	0.95
Nd1—C16^iii^	3.0749 (18)	C6C—C7	1.512 (12)
O3A—N1A	1.218 (6)	O1—C7	1.263 (2)
O4A—N1A	1.228 (4)	O2—C7	1.263 (2)
O5A—C15A	1.317 (5)	O7—C8	1.258 (2)
O5A—H5A	0.84	O8—C8	1.243 (2)
O6A—C15A	1.202 (6)	O8—Nd1^i^	2.4214 (14)
N1A—C4A	1.454 (4)	O9—C16	1.263 (2)
C1A—C2A	1.39	O9—Nd1^iv^	2.4708 (13)
C1A—C6A	1.39	O10—C16	1.261 (2)
C1A—H1AA	0.95	O10—Nd1^ii^	2.4482 (14)
C2A—C3A	1.39	O10—Nd1^iv^	2.9332 (14)
C2A—C15A	1.489 (4)	O11—N2	1.222 (2)
C3A—C4A	1.39	O12—N2	1.235 (2)
C3A—H3AA	0.95	N2—C13	1.468 (2)
C4A—C5A	1.39	C8—C9	1.505 (2)
C5A—C6A	1.39	C9—C14	1.391 (2)
C5A—H5AA	0.95	C9—C10	1.395 (2)
C6A—C7	1.518 (3)	C10—C11	1.393 (2)
O3B—N1B	1.203 (14)	C10—H10A	0.95
O4B—N1B	1.225 (12)	C11—C12	1.395 (2)
O5B—C15B	1.319 (13)	C11—C16	1.496 (2)
O5B—H5B	0.84	C12—C13	1.390 (2)
O6B—C15B	1.166 (12)	C12—H12A	0.95
N1B—C4B	1.469 (11)	C13—C14	1.386 (3)
C1B—C2B	1.39	C14—H14A	0.95
C1B—C6B	1.39	C16—Nd1^iv^	3.0749 (18)
C1B—H1BA	0.95	O1W—H1W1	0.74
C2B—C3B	1.39	O1W—H2W1	0.94
C2B—C15B	1.511 (11)	O2W—H1W2	0.84
C3B—C4B	1.39	O2W—H2W2	0.82
C3B—H3BA	0.95	O3W—H1W3	0.91
C4B—C5B	1.39	O3W—H2W3	0.96
C5B—C6B	1.39	O4WA—H1W4	0.85
C5B—H5BA	0.95	O4WA—H2W4	0.85
C6B—C7	1.480 (9)	O4WB—H3W4	0.75
O3C—N1C	1.239 (16)	O4WB—H4W4	0.79
O4C—N1C	1.223 (15)	O5WA—H1W5	0.85
O5C—C15C	1.294 (15)	O5WA—H2W5	0.85
O5C—H5C	0.90	O5WB—H3W5	0.85
O6C—C15C	1.184 (16)	O5WB—H4W5	0.85
N1C—C4C	1.481 (13)		
			
O7—Nd1—O8^i^	100.76 (5)	C5B—C6B—C1B	120.0
O7—Nd1—O10^ii^	88.82 (5)	C5B—C6B—C7	118.1 (8)
O8^i^—Nd1—O10^ii^	145.95 (5)	C1B—C6B—C7	121.1 (8)
O7—Nd1—O1W	84.42 (5)	O6B—C15B—O5B	124.3 (11)
O8^i^—Nd1—O1W	139.99 (5)	O6B—C15B—C2B	124.3 (10)
O10^ii^—Nd1—O1W	72.98 (5)	O5B—C15B—C2B	111.2 (10)
O7—Nd1—O9^iii^	151.36 (5)	C15C—O5C—H5C	121.2
O8^i^—Nd1—O9^iii^	73.06 (5)	O4C—N1C—O3C	121.9 (17)
O10^ii^—Nd1—O9^iii^	112.18 (5)	O4C—N1C—C4C	121.7 (16)
O1W—Nd1—O9^iii^	83.59 (5)	O3C—N1C—C4C	116.4 (15)
O7—Nd1—O2W	74.02 (5)	C2C—C1C—C6C	120.0
O8^i^—Nd1—O2W	72.51 (6)	C2C—C1C—H1CA	120.0
O10^ii^—Nd1—O2W	141.22 (5)	C6C—C1C—H1CA	120.0
O1W—Nd1—O2W	70.97 (5)	C3C—C2C—C1C	120.0
O9^iii^—Nd1—O2W	77.50 (5)	C3C—C2C—C15C	121.1 (10)
O7—Nd1—O2	123.94 (5)	C1C—C2C—C15C	118.9 (10)
O8^i^—Nd1—O2	72.14 (5)	C4C—C3C—C2C	120.0
O10^ii^—Nd1—O2	75.40 (5)	C4C—C3C—H3CA	120.0
O1W—Nd1—O2	136.59 (5)	C2C—C3C—H3CA	120.0
O9^iii^—Nd1—O2	81.61 (5)	C3C—C4C—C5C	120.0
O2W—Nd1—O2	142.83 (6)	C3C—C4C—N1C	117.9 (11)
O7—Nd1—O1	73.37 (5)	C5C—C4C—N1C	122.1 (11)
O8^i^—Nd1—O1	70.84 (5)	C6C—C5C—C4C	120.0
O10^ii^—Nd1—O1	81.06 (5)	C6C—C5C—H5CA	120.0
O1W—Nd1—O1	146.05 (5)	C4C—C5C—H5CA	120.0
O9^iii^—Nd1—O1	127.22 (5)	C5C—C6C—C1C	120.0
O2W—Nd1—O1	124.29 (5)	C5C—C6C—C7	115.6 (10)
O2—Nd1—O1	51.38 (4)	C1C—C6C—C7	123.6 (10)
O7—Nd1—C7	99.34 (5)	O6C—C15C—O5C	128.0 (16)
O8^i^—Nd1—C7	65.51 (5)	O6C—C15C—C2C	118.1 (14)
O10^ii^—Nd1—C7	80.77 (5)	O5C—C15C—C2C	113.8 (14)
O1W—Nd1—C7	153.43 (5)	C7—O1—Nd1	90.75 (11)
O9^iii^—Nd1—C7	102.98 (5)	C7—O2—Nd1	93.16 (11)
O2W—Nd1—C7	135.46 (6)	C8—O7—Nd1	171.21 (13)
O2—Nd1—C7	25.94 (5)	C8—O8—Nd1^i^	136.37 (12)
O1—Nd1—C7	25.99 (5)	C16—O9—Nd1^iv^	106.33 (11)
O7—Nd1—O10^iii^	146.58 (4)	C16—O10—Nd1^ii^	160.39 (12)
O8^i^—Nd1—O10^iii^	112.58 (4)	C16—O10—Nd1^iv^	84.27 (11)
O10^ii^—Nd1—O10^iii^	64.92 (5)	Nd1^ii^—O10—Nd1^iv^	115.08 (5)
O1W—Nd1—O10^iii^	68.84 (4)	O11—N2—O12	124.08 (17)
O9^iii^—Nd1—O10^iii^	47.27 (4)	O11—N2—C13	118.38 (17)
O2W—Nd1—O10^iii^	113.16 (4)	O12—N2—C13	117.54 (16)
O2—Nd1—O10^iii^	71.17 (4)	O2—C7—O1	122.11 (17)
O1—Nd1—O10^iii^	119.02 (4)	O2—C7—C6B	120.1 (5)
C7—Nd1—O10^iii^	96.50 (5)	O1—C7—C6B	117.7 (5)
O7—Nd1—C16^iii^	159.88 (5)	O2—C7—C6C	125.0 (7)
O8^i^—Nd1—C16^iii^	92.18 (5)	O1—C7—C6C	112.3 (7)
O10^ii^—Nd1—C16^iii^	88.96 (5)	O2—C7—C6A	117.2 (2)
O1W—Nd1—C16^iii^	75.82 (5)	O1—C7—C6A	120.5 (2)
O9^iii^—Nd1—C16^iii^	23.21 (5)	O2—C7—Nd1	60.91 (9)
O2W—Nd1—C16^iii^	95.64 (5)	O1—C7—Nd1	63.26 (10)
O2—Nd1—C16^iii^	74.63 (5)	C6B—C7—Nd1	166.1 (8)
O1—Nd1—C16^iii^	125.93 (4)	C6C—C7—Nd1	171.4 (11)
C7—Nd1—C16^iii^	100.03 (5)	C6A—C7—Nd1	160.6 (3)
O10^iii^—Nd1—C16^iii^	24.08 (4)	O8—C8—O7	125.51 (17)
C15A—O5A—H5A	109.5	O8—C8—C9	117.18 (16)
O3A—N1A—O4A	124.8 (4)	O7—C8—C9	117.30 (16)
O3A—N1A—C4A	118.3 (3)	C14—C9—C10	120.32 (16)
O4A—N1A—C4A	116.9 (4)	C14—C9—C8	120.22 (15)
C2A—C1A—C6A	120.0	C10—C9—C8	119.44 (16)
C2A—C1A—H1AA	120.0	C11—C10—C9	120.31 (16)
C6A—C1A—H1AA	120.0	C11—C10—H10A	119.8
C3A—C2A—C1A	120.0	C9—C10—H10A	119.8
C3A—C2A—C15A	119.6 (3)	C10—C11—C12	120.42 (16)
C1A—C2A—C15A	120.3 (3)	C10—C11—C16	119.21 (16)
C4A—C3A—C2A	120.0	C12—C11—C16	120.37 (16)
C4A—C3A—H3AA	120.0	C13—C12—C11	117.41 (16)
C2A—C3A—H3AA	120.0	C13—C12—H12A	121.3
C3A—C4A—C5A	120.0	C11—C12—H12A	121.3
C3A—C4A—N1A	120.1 (3)	C14—C13—C12	123.58 (16)
C5A—C4A—N1A	119.9 (3)	C14—C13—N2	118.54 (16)
C4A—C5A—C6A	120.0	C12—C13—N2	117.84 (16)
C4A—C5A—H5AA	120.0	C13—C14—C9	117.75 (16)
C6A—C5A—H5AA	120.0	C13—C14—H14A	121.1
C5A—C6A—C1A	120.0	C9—C14—H14A	121.1
C5A—C6A—C7	119.4 (2)	O10—C16—O9	122.04 (17)
C1A—C6A—C7	120.6 (2)	O10—C16—C11	119.94 (16)
O6A—C15A—O5A	124.1 (4)	O9—C16—C11	118.02 (16)
O6A—C15A—C2A	122.3 (3)	O10—C16—Nd1^iv^	71.65 (10)
O5A—C15A—C2A	113.5 (4)	O9—C16—Nd1^iv^	50.45 (9)
C15B—O5B—H5B	109.5	C11—C16—Nd1^iv^	167.91 (13)
O3B—N1B—O4B	123.1 (13)	Nd1—O1W—H1W1	118.6
O3B—N1B—C4B	118.3 (12)	Nd1—O1W—H2W1	125.6
O4B—N1B—C4B	118.5 (12)	H1W1—O1W—H2W1	102.4
C2B—C1B—C6B	120.0	Nd1—O2W—H1W2	123.6
C2B—C1B—H1BA	120.0	Nd1—O2W—H2W2	127.6
C6B—C1B—H1BA	120.0	H1W2—O2W—H2W2	106.9
C3B—C2B—C1B	120.0	H1W3—O3W—H2W3	115.1
C3B—C2B—C15B	119.6 (8)	H1W4—O4WA—H2W4	107.4
C1B—C2B—C15B	120.3 (8)	H1W4—O4WA—H3W4	54.4
C2B—C3B—C4B	120.0	H2W4—O4WA—H3W4	64.4
C2B—C3B—H3BA	120.0	H1W4—O4WB—H2W4	97.0
C4B—C3B—H3BA	120.0	H1W4—O4WB—H3W4	60.6
C3B—C4B—C5B	120.0	H2W4—O4WB—H3W4	123.4
C3B—C4B—N1B	118.5 (9)	H1W4—O4WB—H4W4	164.0
C5B—C4B—N1B	121.5 (9)	H2W4—O4WB—H4W4	79.4
C6B—C5B—C4B	120.0	H3W4—O4WB—H4W4	108.3
C6B—C5B—H5BA	120.0	H1W5—O5WA—H2W5	107.4
C4B—C5B—H5BA	120.0	H3W5—O5WB—H4W5	107.7
			
C6A—C1A—C2A—C3A	0.0	C5B—C6B—C7—Nd1	73 (2)
C6A—C1A—C2A—C15A	−176.5 (5)	C1B—C6B—C7—Nd1	−96 (2)
C1A—C2A—C3A—C4A	0.0	C5C—C6C—C7—O2	−25.6 (17)
C15A—C2A—C3A—C4A	176.6 (5)	C1C—C6C—C7—O2	164.7 (10)
C2A—C3A—C4A—C5A	0.0	C5C—C6C—C7—O1	163.1 (9)
C2A—C3A—C4A—N1A	−177.8 (4)	C1C—C6C—C7—O1	−6.7 (19)
O3A—N1A—C4A—C3A	167.1 (4)	C5C—C6C—C7—C6B	33 (8)
O4A—N1A—C4A—C3A	−13.6 (6)	C1C—C6C—C7—C6B	−137 (10)
O3A—N1A—C4A—C5A	−10.7 (6)	C5C—C6C—C7—C6A	36 (3)
O4A—N1A—C4A—C5A	168.6 (4)	C1C—C6C—C7—C6A	−134 (5)
C3A—C4A—C5A—C6A	0.0	C5A—C6A—C7—O2	3.6 (4)
N1A—C4A—C5A—C6A	177.8 (4)	C1A—C6A—C7—O2	−178.3 (3)
C4A—C5A—C6A—C1A	0.0	C5A—C6A—C7—O1	178.6 (2)
C4A—C5A—C6A—C7	178.2 (5)	C1A—C6A—C7—O1	−3.2 (5)
C2A—C1A—C6A—C5A	0.0	C5A—C6A—C7—C6B	−117 (7)
C2A—C1A—C6A—C7	−178.2 (5)	C1A—C6A—C7—C6B	61 (7)
C3A—C2A—C15A—O6A	−174.7 (5)	C5A—C6A—C7—C6C	−122 (4)
C1A—C2A—C15A—O6A	1.8 (8)	C1A—C6A—C7—C6C	56 (4)
C3A—C2A—C15A—O5A	2.6 (6)	C5A—C6A—C7—Nd1	82.8 (6)
C1A—C2A—C15A—O5A	179.2 (4)	C1A—C6A—C7—Nd1	−99.0 (5)
C6B—C1B—C2B—C3B	0.0	O7—Nd1—C7—O2	−162.80 (11)
C6B—C1B—C2B—C15B	−177.4 (17)	O8^i^—Nd1—C7—O2	99.56 (12)
C1B—C2B—C3B—C4B	0.0	O10^ii^—Nd1—C7—O2	−75.54 (11)
C15B—C2B—C3B—C4B	177.4 (17)	O1W—Nd1—C7—O2	−66.56 (17)
C2B—C3B—C4B—C5B	0.0	O9^iii^—Nd1—C7—O2	35.26 (12)
C2B—C3B—C4B—N1B	177.8 (12)	O2W—Nd1—C7—O2	120.53 (12)
O3B—N1B—C4B—C3B	−170.2 (14)	O1—Nd1—C7—O2	−164.04 (19)
O4B—N1B—C4B—C3B	7.7 (19)	O10^iii^—Nd1—C7—O2	−12.32 (12)
O3B—N1B—C4B—C5B	8(2)	C16^iii^—Nd1—C7—O2	11.75 (12)
O4B—N1B—C4B—C5B	−174.5 (13)	O7—Nd1—C7—O1	1.25 (12)
C3B—C4B—C5B—C6B	0.0	O8^i^—Nd1—C7—O1	−96.39 (12)
N1B—C4B—C5B—C6B	−177.8 (12)	O10^ii^—Nd1—C7—O1	88.51 (11)
C4B—C5B—C6B—C1B	0.0	O1W—Nd1—C7—O1	97.48 (15)
C4B—C5B—C6B—C7	−169.7 (17)	O9^iii^—Nd1—C7—O1	−160.70 (11)
C2B—C1B—C6B—C5B	0.0	O2W—Nd1—C7—O1	−75.42 (13)
C2B—C1B—C6B—C7	169.3 (17)	O2—Nd1—C7—O1	164.04 (19)
C3B—C2B—C15B—O6B	178.9 (11)	O10^iii^—Nd1—C7—O1	151.72 (11)
C1B—C2B—C15B—O6B	−4(2)	C16^iii^—Nd1—C7—O1	175.79 (11)
C3B—C2B—C15B—O5B	−6.3 (17)	O7—Nd1—C7—C6B	99 (2)
C1B—C2B—C15B—O5B	171.1 (13)	O8^i^—Nd1—C7—C6B	1(2)
C6C—C1C—C2C—C3C	0.0	O10^ii^—Nd1—C7—C6B	−174 (2)
C6C—C1C—C2C—C15C	179.4 (19)	O1W—Nd1—C7—C6B	−165 (2)
C1C—C2C—C3C—C4C	0.0	O9^iii^—Nd1—C7—C6B	−63 (2)
C15C—C2C—C3C—C4C	−179 (2)	O2W—Nd1—C7—C6B	22 (2)
C2C—C3C—C4C—C5C	0.0	O2—Nd1—C7—C6B	−98 (2)
C2C—C3C—C4C—N1C	178 (2)	O1—Nd1—C7—C6B	98 (2)
O4C—N1C—C4C—C3C	5(3)	O10^iii^—Nd1—C7—C6B	−111 (2)
O3C—N1C—C4C—C3C	−176 (2)	C16^iii^—Nd1—C7—C6B	−86 (2)
O4C—N1C—C4C—C5C	−177 (2)	O7—Nd1—C7—C6A	107.5 (5)
O3C—N1C—C4C—C5C	2(3)	O8^i^—Nd1—C7—C6A	9.9 (5)
C3C—C4C—C5C—C6C	0.0	O10^ii^—Nd1—C7—C6A	−165.2 (5)
N1C—C4C—C5C—C6C	−178 (2)	O1W—Nd1—C7—C6A	−156.2 (5)
C4C—C5C—C6C—C1C	0.0	O9^iii^—Nd1—C7—C6A	−54.4 (5)
C4C—C5C—C6C—C7	−170 (2)	O2W—Nd1—C7—C6A	30.9 (5)
C2C—C1C—C6C—C5C	0.0	O2—Nd1—C7—C6A	−89.7 (5)
C2C—C1C—C6C—C7	169 (2)	O1—Nd1—C7—C6A	106.3 (5)
C3C—C2C—C15C—O6C	149.3 (18)	O10^iii^—Nd1—C7—C6A	−102.0 (5)
C1C—C2C—C15C—O6C	−30 (3)	C16^iii^—Nd1—C7—C6A	−77.9 (5)
C3C—C2C—C15C—O5C	−28 (3)	Nd1^i^—O8—C8—O7	4.0 (3)
C1C—C2C—C15C—O5C	152.6 (16)	Nd1^i^—O8—C8—C9	−175.30 (12)
O7—Nd1—O1—C7	−178.71 (12)	O8—C8—C9—C14	−156.86 (17)
O8^i^—Nd1—O1—C7	73.22 (11)	O7—C8—C9—C14	23.8 (2)
O10^ii^—Nd1—O1—C7	−87.26 (11)	O8—C8—C9—C10	24.8 (2)
O1W—Nd1—O1—C7	−127.43 (12)	O7—C8—C9—C10	−154.54 (17)
O9^iii^—Nd1—O1—C7	23.86 (13)	C14—C9—C10—C11	−3.1 (3)
O2W—Nd1—O1—C7	124.75 (12)	C8—C9—C10—C11	175.22 (15)
O2—Nd1—O1—C7	−8.85 (11)	C9—C10—C11—C12	4.5 (3)
O10^iii^—Nd1—O1—C7	−32.57 (12)	C9—C10—C11—C16	−174.87 (16)
C16^iii^—Nd1—O1—C7	−5.12 (13)	C10—C11—C12—C13	−1.6 (3)
O7—Nd1—O2—C7	20.60 (13)	C16—C11—C12—C13	177.77 (16)
O8^i^—Nd1—O2—C7	−70.53 (12)	C11—C12—C13—C14	−2.8 (3)
O10^ii^—Nd1—O2—C7	99.01 (12)	C11—C12—C13—N2	179.38 (16)
O1W—Nd1—O2—C7	143.33 (11)	O11—N2—C13—C14	160.22 (19)
O9^iii^—Nd1—O2—C7	−145.35 (12)	O12—N2—C13—C14	−19.7 (3)
O2W—Nd1—O2—C7	−89.17 (13)	O11—N2—C13—C12	−21.9 (3)
O1—Nd1—O2—C7	8.87 (11)	O12—N2—C13—C12	158.19 (17)
O10^iii^—Nd1—O2—C7	167.05 (12)	C12—C13—C14—C9	4.1 (3)
C16^iii^—Nd1—O2—C7	−168.00 (12)	N2—C13—C14—C9	−178.07 (16)
Nd1—O2—C7—O1	−16.8 (2)	C10—C9—C14—C13	−1.1 (3)
Nd1—O2—C7—C6B	164.1 (9)	C8—C9—C14—C13	−179.40 (16)
Nd1—O2—C7—C6C	172.6 (13)	Nd1^ii^—O10—C16—O9	173.8 (3)
Nd1—O2—C7—C6A	158.1 (3)	Nd1^iv^—O10—C16—O9	2.76 (17)
Nd1—O1—C7—O2	16.47 (19)	Nd1^ii^—O10—C16—C11	−5.2 (5)
Nd1—O1—C7—C6B	−164.4 (9)	Nd1^iv^—O10—C16—C11	−176.24 (15)
Nd1—O1—C7—C6C	−171.9 (12)	Nd1^ii^—O10—C16—Nd1^iv^	171.0 (4)
Nd1—O1—C7—C6A	−158.3 (3)	Nd1^iv^—O9—C16—O10	−3.4 (2)
C5B—C6B—C7—O2	−17.0 (11)	Nd1^iv^—O9—C16—C11	175.62 (12)
C1B—C6B—C7—O2	173.5 (8)	C10—C11—C16—O10	145.14 (17)
C5B—C6B—C7—O1	163.9 (5)	C12—C11—C16—O10	−34.2 (2)
C1B—C6B—C7—O1	−5.6 (16)	C10—C11—C16—O9	−33.9 (2)
C5B—C6B—C7—C6C	−143 (9)	C12—C11—C16—O9	146.75 (17)
C1B—C6B—C7—C6C	47 (8)	C10—C11—C16—Nd1^iv^	−17.6 (7)
C5B—C6B—C7—C6A	45 (7)	C12—C11—C16—Nd1^iv^	163.1 (5)
C1B—C6B—C7—C6A	−124 (8)		

Symmetry codes: (i) −*x*, −*y*+1, −*z*+1; (ii) −*x*, −*y*+2, −*z*+1; (iii) *x*+1, *y*−1, *z*; (iv) *x*−1, *y*+1, *z*.

### Hydrogen-bond geometry (Å, °)

**Table:**

*D*—H···*A*	*D*—H	H···*A*	*D*···*A*	*D*—H···*A*
O5A—H5A···O3W^v^	0.84	1.76	2.596 (5)	178
O1W—H1W1···O2^vi^	0.74	1.99	2.731 (3)	175
O1W—H2W1···O4WA	0.94	2.00	2.849 (4)	149
O2W—H1W2···O4WA	0.84	1.86	2.667 (3)	159
O2W—H2W2···O1^i^	0.82	2.06	2.874 (3)	172
O3W—H1W3···O11^vii^	0.91	2.56	3.246 (3)	132
O3W—H1W3···O12^vii^	0.91	2.05	2.902 (3)	156
O3W—H2W3···O9^iii^	0.96	1.76	2.688 (2)	162
O4WA—H2W4···O3W	0.85	2.51	3.118 (6)	130
C12—H12A···O1^ii^	0.95	2.41	3.351 (2)	169

Symmetry codes: (v) *x*−1, *y*, *z*+1; (vi) −*x*+1, −*y*+1, −*z*+1; (i) −*x*, −*y*+1, −*z*+1; (vii) *x*, *y*−1, *z*; (iii) *x*+1, *y*−1, *z*; (ii) −*x*, −*y*+2, −*z*+1.
